# Cannabidiol effects on behaviour and immune gene expression in zebrafish (*Danio rerio*)

**DOI:** 10.1371/journal.pone.0200016

**Published:** 2018-07-31

**Authors:** Hannah M. Jensen, Rozalia Korbut, Per W. Kania, Kurt Buchmann

**Affiliations:** Department of Veterinary and Animal Sciences, Faculty of Health and Medical Sciences, University of Copenhagen, Frederiksberg C., Denmark; Deakin School of Medicine, AUSTRALIA

## Abstract

Various preparations and extracts of the plant *Cannabis sativa* (family cannabaceae) are used as herbal medicinal drugs against a series of disorders but the plant contains a wide series of pharmacologically active components which may confound evaluation of drug effects. In order to differentiate specific effects of the individual constituents on specific functions in the organism we advocate for controlled studies on specified constituents and their impact on the vertebrate organism. One of the dominating *Cannabis* constituents, delta(9)-tetrahydrocannabinol (THC), has previously been studied in depth whereas information on another main ingredient cannabidiol (CBD) is limited. We have performed a controlled study on CBD and its effect using an experimental zebrafish model. CDB treatment of zebrafish for 30 min affected mobility of the fish by decreasing swimming speed and swimming distance. In addition, out of 23 immune related genes studied it was shown that expression of two genes *il1b* and *il17a/f2* were up-regulated and four genes, *tgfba*, *ighm*, *cd4-1*, and *s100a10b* were significantly down-regulated following CBD treatment. The study indicated that CBD affects motility and immunity of the vertebrate host.

## Introduction

Various preparations and extracts of *Cannabis sativa* (family cannabaceae), used either for medical or recreational purposes, have been reported to affect the level of stress, anxiety, depression and even induce euphoria [1]. However, *C*. *sativa* is composed of 85 different constituents, termed cannabinoids [2], and in order to achieve full understanding of pharmacological effects of this plant on the vertebrate organism it is necessary to evaluate each of the individual components. Cannabidiol (CBD) is one of the major constituents in *C*. *sativa* but information on its precise action mechanisms is limited. CBD is generally considered a non-psychoactive compound but has been reported to reduce epileptic seizures in a rat model [3][4], alter behaviour and anxiety in mice lacking the CBD receptor CB_1_R [1], possess anti-oxidative and anti-apoptotic properties in rat cells [5], exert anti-inflammatory effects in mice [6][7], induce apoptosis in immortalized and primary glial cells [5] [8], and in human glioma cells [9][10][11][12]. CBD has low affinity for cannabinoid receptors, but antagonizes CB_1_R/CB_2_R agonists whereby CBD may inhibit immune cell migration and counteract inflammation [13]. With regard to the immune regulating effect of CBD it is noteworthy that CBD has been shown to inhibit production of il10 in human T-cells, increase cxcl8a, MIP-1a and MIP-1b levels in an eosinophilic leukemia cell line and inhibit cxcl8a production in a HTLV-1 positive B cell line [14]. The scattered evidence of important physiological CBD effects call for additional basic studies on CBD effects in vertebrates and we have chosen a zebrafish model for elucidation of behavioural and immunological impacts. The zebrafish (*Danio rerio*) is a vertebrate organism with high resemblance to humans with regard to the genome (approximately 70%) [15], biochemical pathways [16] and response to neurotropic drugs [17] and therefore we selected zebrafish as a suitable model in this study with focus on behaviour and immune gene expression in CBD treated zebrafish. By exposing adult wildtype zebrafish to purified CBD in a fixed concentration and subsequently measuring behavioural changes (swimming speed and distance) and recording expression of an array of central immune genes we demonstrate that CBD both affects locomotory abilities, inflammation and immunity.

## Materials and methods

### Ethic statement

All experiments were conducted under license no. 2013-15-2934-00867 approved by the Animal Experiments Inspectorate under the Danish Ministry of Environment and Food, Agriculture and Fisheries, Denmark.

### Animals and housing

Wild–type zebrafish (*Danio rerio*) of own in-house breed were reared in a recirculated system (10% daily water exchange) (Aquaschwarz, Germany) at 28°C with a light-darkness cycle of 14h L: 10h D, pH 7.4 and conductivity 550 μS at our experimental zebrafish facility at the University of Copenhagen, Frederiksberg, Denmark. Fish were fed live *Artemia* and pelleted dry feed (ZM Fish Food, England) twice a day. Adult fish (age 6 months) were used and when experimentally exposed in water we applied sterile filtered (0.2 μm) (Sartorius) fish tank water.

### Chemicals used

CBD (stock solution 10 mg/mL in 1 ml ethanol, Sigma-Aldrich cat. number 90899-1ML) was used to obtain the desired concentration of 40 mg/L by adding 0.2 mL CBD stock solution to 49.8 mL of sterile-filtered zebrafish system water (final volume 50 mL). A corresponding solvent control (ethanol solution without CBD) was produced by adding 0.2 mL 96% ethanol (Plum A/S cat. number 201146) to 49.8 mL sterile-filtered system water (0.4% ethanol). Similarly filtered system water served as pure water control (no solvent, no CBD).

### Experimental design when studying gene expression

A total of 30 zebrafish was divided into three experimental groups (each containing 2x5 fish): 1) pure water control (facility water), 2) solvent control (0.4% ethanol) and 3) CBD (CBD in 0.4% ethanol in water). Before experimental start each duplicate group was acclimatized for 1h in a 1.8L tank containing water from the facility whereafter individual fish were placed separately in a well (6-well plate, Greiner bio-one, CELLSTAR) containing 10 ml of the test solution. After 30 min exposure (water, ethanol or CBD) fish were euthanized by immersion into an overdose (30 mg/L) etomidate (Sigma-Aldrich, Denmark) solution, subsequently dissected under a stereomicroscope (Leica MZ12.5, Leica Denmark) and internal organs (intestine, liver, spleen) transferred to RNAlater (Sigma-Aldrich, Denmark). Preserved material was kept at 4°C for 24h and then frozen at -20°C until processing for gene expression studies.

### Experimental design when studying behaviour

A total of 30 adult zebrafish, divided into three experimental groups (each comprising 2x5 fish), was observed following exposure (25°C) to 1) sterile filtered system water (water control), 2) 0.4% ethanol solution (solvent control) and 3) 40 mg/L CBD solution (with 0.4% ethanol as solvent). Video tracking and swim track analysis of adult zebrafish behaviour was performed by use of the EthoVision XT 10.1 system (Noldus Information Technologies, The Netherlands). Individual fish were exposed in separate wells (containing 10 ml test solution) in a 6-well cell culture plate (Greiner bio-one, CELLSTAR. Each fish was separately placed in a well, exposed to the solution for 30 minutes, and transferred to the observation tank (tank dimensions: 24.5x15x15cm) containing 3.12 L filtered system water (water level 8.5 cm). The fish were acclimatized for 3–4 minutes in the tank before 3 min video-recording. Fish movements were followed with a top view camera recording movements over the ground area, and a side view camera recording movements in the water column. The video tracking files were stored for subsequent analysis (Noldus Information Technologies, The Netherlands).

### Gene expression

#### RNA purification and cDNA synthesis

Total RNA was extracted using the GenEluteTM mammalian total RNA kit (Sigma-Aldrich, Denmark) according to the manufacturer’s instruction. In brief, zebrafish organ samples were incubated in 300 μL lysis buffer containing 2-mercaptoethanol and sonicated using a sonic dismembrator (Bie&Berntsen A/S). Genomic DNA was removed by DNase I (ThermoFisher Scientific, Denmark) treatment and the chelating agent EDTA (ThermoFisher Scientific, Denmark) was added. RNA concentration was measured using a NanoDrop 2000 spectrophotometer (Saveen & Werner ApS, Denmark) and quality assessed by 1.5% ethidium bromide stained-agarose gel electrophoresis (Cleaver Scientific Ltd, UK). RNA samples were stored at -80°C until cDNA synthesis performed in Biometra T3 thermocycler (Fisher Scientific, Germany) using 1000ng of RNA, Oligo d(T)16 primers and MultiScribeTM reverse transcription reagents (Applied Biosystems, Denmark). A reaction volume of 20 μL was used at following conditions: (25°C/10min, 37°C/60min, 95°C/5min) and subsequently the reaction product was diluted 10 times into 200 μL with RNase-free water (Invitrogen, Denmark) and stored at -20°C.

#### Quantitative Real-Time PCR (qPCR)

Gene expression levels were assessed applying a TagMan probe-based assay and an AriaMx Real-Time PCR machine (Agilent Technologies, AH diagnostics A/S, Denmark). Genes investigated encoded cytokines (*il1b*, *il13*, *il4*, *il6*, *cxcl8a*, *il10*, *il12a*, *il17a/f1*, *il17a/f2*, *il17a/f3*, *il22*, *ifng1*, *tgfba*, *tnfa*), acute phase proteins (*saa*) cellular receptors (*cd4-1*, *cd8a*), immunoglobulins (*ighm*, *ighz*), matrix metalloproteinase-9 (*mmp9*), transcription factors (*gata3*, *tbx21*), calcium binding protein *s100a10b* and reference genes *acbt2*, *EF-1α* and *rpl13a*. Primers and probes (Table 1) were designed as described by Haarder et al. [18]. The reaction volume 12.5 μL included: 2.5μL of cDNA, 6.25 μL Brilliant III Ultra-Fast QPCR Master Mix (Agilent Technologies, Denmark), 1μL of forward primer and 2.75 μL of RNase-free water. Reaction conditions: 95°C/3 min followed by 40 cycles of denaturation at 95°C for 5 sec combined with annealing/elongation step at 60°C for 15 sec. Negative controls included RT minus and mastermix plus water.

**Table 1:** Primers and probes used for gene expression analysis. TagMan probe based qPCR assays according to Haarder et al. (2016). All the assays were optimized to have primer-annealing temperature at 60°C and amplification efficiencies of 100% ±5%. The three genes *acbt2*, *eelf1a1l1* and *rpl13a* served as reference genes. All the nucleotides are oriented from the 3’end towards the 5’end. The probes were labelled with FAM at 3’end and with BQ1 at the 5’end. Genes names according to ZFIN.org.

Gene	Forward primer	Reverse primer	Probe	GenBank Acc.no.
*acbt2*	*CCATCCTTCTTGGGTATGGA*	*ACAGGTCCTTACGGATGTCG*	*TGCGGTATCCACGAGACCACC*	*BC154531*
*cd4-1*	*CCAGACTGGAGCCAAGAGAC*	CAGTGCAGCTCCACATCACT	*CCTGCGCACAGTCAGGGAAA*	*EF601915*
*cd8a*	*TCGGAGGTTGTGGACTTTTC*	*TAATGGTGGGGACATCGTCT*	*CGTTCTGCTGATCGCAACCA*	*BC114236*
*cxcl8a*	GATCTGTCTGGACCCCTCTG	GGGCATTCATGGTTTTCTGT	CCATGGGTTAAGAAGATCATTGATAGG	XM_001342570
*eelf1a1l1*	*GAACGACCCACCCATGGAGG*	*TGATGACCTGAGCGTTGAAG*	*GAACGACCCACCCATGGAGG*	*AY422992*
*gata3*	*TACGTGTCCCGCTTAAAACC*	*TGAAGGGGCAATGAAGAAAG*	*GGCCTTCACTTTCGCCTGCT*	*BC162389*
*Ifng1*	TATGGGCGATCAAGGAAAAC	CTTTAGCCTGCCGTCTCTTG	CGATCGTCCAGCGAAAGGCT	AB158361
*ighm*	*TGCAGTTCTGGTTCTGATGG*	*TGCACAAAATCGCTCAAATC*	*AATCACCCTCGGCTGCTTGG*	*AY643753*
*ighz*	*ATTGGATGTCTGGCCTCTGA*	*AATGCTGGGTGACGTTTTTC*	*TGCACAAAATCGCTCAAATC*	*AY643750*
*il1b*	*CGCTCCACATCTCGTACTCA*	*ATACGCGGTGCTGATAAACC*	*GAAGGAGACCGGCAGCTCCA*	BC098597
*Il4*	AACTCTCTGCCAAGCAGGAA	AAACGCTGCAGTTTCCAGTC	GAGACAGCTGAATGCTTATGCAGCA	AB375405
*il 6*	AGACCGCTGCCTGTCTAAAA	CAACTTCTCCAGCGTGATGA	TCCGCATGGACTCGCAAGAC	JN698962
*il10*	*CTTGCCAAAATCCCTTTGAA*	ATCAAGCTCCCCCATAGCTT	TGAAAAGATGAAGGAAAAGGGGG	*BC163038*
*il12a*	*AGCAGGACTTGTTTGCTGGT*	*TCCACTGCGCTGAAGTTAGA*	*TAACTCGTCCTGCTCGGCCC*	*AB183001*
*Il13*	GTCAGGCTGAGGAGGAGATG	AGCAGCGTGACTCCTGATCT	CTGGCCTGTCCGGTGTCAAA	AB375404
*il17a/f1*	*GCTTTCTTATGGTGGCTTGC*	*GCCGGTATGAATGATCTGCT*	*ATCATTCGGTGCTGAGGGGG*	*AB195256*
*il17a/f2*	*TCAATCTGAGGACGGAAAGG*	*GCTCCATCTCCTGTTTCAGC*	*CATCTTGCCCACTGGTGTGGA*	*NM_001020798*
*il17a/f3*	*GGCTGCACATGTGTTTTACC*	*CAATGTGATTCGTTTTCAGGCT*	*CAGCGCCTATAGAAATAATCACTCCG*	*AB195260*
*il 22*	*GGATTACGCCAAAGGTGAAA*	*CGAGCACAGCAAAGCAATAA*	*CGACATCGAGGAACAACGGTG*	*NM_001020792*
*mmp9*	ACAGGGAGACGCTCATTTTG	TGTTCCCTCAAACAGGAAGG	CGGTAATGCTGAGGGTGCAATGTGT	BC160656
*rpl13a*	TCCCAGCTGCTCTCAAGATT	ACTTCCAGCCAACTTCATGG	CACACGCAAATTTGCCCTGC	NM_212784
*s100a10b*	GCAGAGGGGAACTCATCAAC	CCCACCACAAGAGACACAAA	CAGCTGATGGAGAAAGAGCTGGC	BC163954
*saa*	CTTGCTGTGCTGGTGATGTT	CTTCCAATTGGCCTCTTTCA	CGCTGGAGGTGCAAAGGACA	BC081487
*tbx21*	AAATCCAGGAGCATGGACAG	TGAGACTGGATGTGGGTTTG	TGGTTTTCCTATTGGAAGGCGG	AM942761
*tgfba*	TGCGCAAGCTTTACATTGAC	AGGACCCCATGCAGTAGTTG	TGGATCCACAAGCCCAAGGG	AY178450
*tnfa*	GCGCTTTTCTGAATCCTACG	AAGTGCTGTGGTCGTGTCTG	TGCACGCAGGAGCCTGAATC	AY427649

### Data analyses and statistics

For relative gene expression determination, the 2^−ΔΔC^_T_ method by Livak & Schmittgen [19] was used. The geometric mean of the ethanol groups served as control group for the CBD group due to the use of ethanol as a solvent for CBD. A more comprehensive resume of all the gene expression results is available in the Supporting Information (S1 Table). In order to reveal the effect of ethanol itself, the solvent for CBD, S1 Table includes results obtained by using the water groups as control groups.

An average of three reference genes (*acbt2*, elongation factor (*eelf1a1l1*), ribosomal *rpl13a)* was applied for normalization. Data were compared for all treatments and related to both water and ethanol controls using Student’s t-test and a probability level of 5%. The qualitative regulations were assessed by a Mann-Whitney *U*-test applying the same probability level.

In order to obtain a 3D spatial reconstruction of fish movement, fish movement data were subjected to Track3D (Noldus Information Technologies, The Netherlands) and subsequently analysed in RapidMiner Studio 6.3 (RapidMiner, USA). Raw data was used for statistical analysis. To assess whether the behaviour in the different experimental groups were statistically different from each other, a one-tailed Student’s t-test was used to compare mean swimming speed and distance using a 5% probability level.

## Results

### Behaviour of zebrafish exposed to CBD

**Fig 1:**
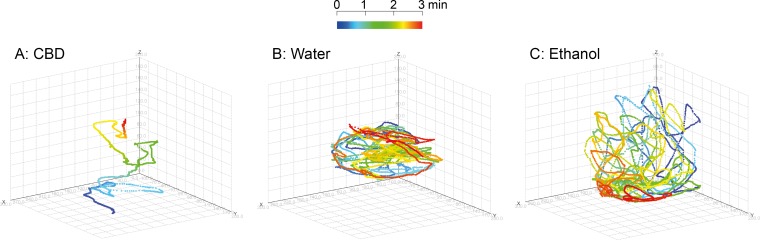
Examples of 3D-tracking of fish movements. Three examples illustrating the different movement patterns of zebrafish following exposure to water, ethanol and CBD. Different colours indicates the time intervals during the study period of 3 min as indicated by the top bar, starting at 0 min with dark blue via green and yellow ending with dark red at 3 min. A) CBD treated zebrafish, B) System water treated zebrafish and C) Ethanol treated zebrafish.

**Fig 2:**
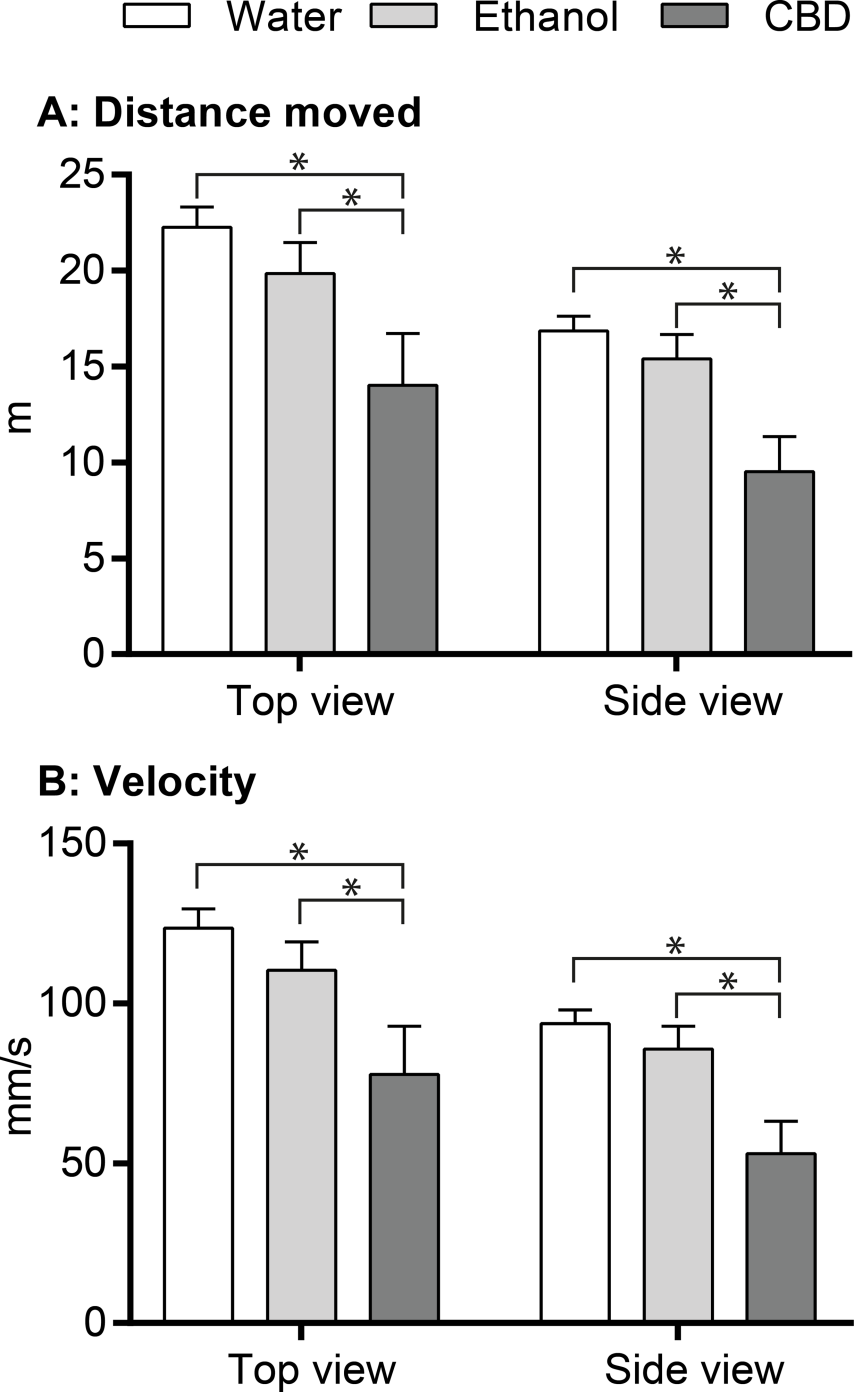
Swimming behaviour of zebrafish reflected by the mean distances and the mean swimming velocities after 30 min exposure to either CBD dissolved in ethanol, ethanol alone or water. The top views and the side views are treated individually. *: p<0.05, Student’s t-test.

Following 30 min exposure to either filtered system water (water control), ethanol solution (ethanol control) or CBD solution, zebrafish were tested with regard to swimming performance in a test tank for 3 min. Examples of 3D tracks of zebrafish movements under different conditions are shown in Fig 1. Statistically significant differences with regard to swimming velocity and total swimming distance were found between water control fish and CBD-exposed fish and between ethanol control fish and CBD-exposed fish. Thus during the 3 min observation, the top view revealed the CBD treated fish moved with 63% and 71% of the velocity (Table 2) of water and ethanol control fish, respectively; the same numbers from the side view were 57% and 62%, respectively. No difference in behaviour between fish kept in water and fish exposed to ethanol (solvent control) were recorded (Table 2 and Fig 2).

**Table 2:** Behavioural effects of CBD on zebrafish. The fish were exposed for 30 min to either CBD dissolved in ethanol, ethanol alone or water. The behaviour, reflected as swimming distance and swimming velocity, was recorded by top and side view cameras.

		Ratio	t-test		Ratio	t-test		Ratio	t-test
		Ethanol vs Water			CBD vs Water			CBD vs Ethanol	
Top view	Velocity	1.12		0.11	0.63		0.01*	0.71	0.04*
	Distance	0.89		0.11	0.63		0.01*	0.71	0.04*
Side view	Velocity	0.92		0.18	0.57		<0.01*	0.62	0.01*
	Distance	0.91		0.17	0.57		<0.01*	0.62	0.01*
		Water			Ethanol			CBD	
Top view vs Side view	Velocity	1.32		<0.01*	1.29		0.02*	1.47	0.09
	Distance	1.32		<0.01*	1.29		0.02*	1.47	0.09

*: p<0.05, Student’s t-test.

Upper part of the table (above the dotted line): comparison of treatment groups. Lower part of the table (under the dotted line): comparison between top and side views.

### Gene expression analyses

**Fig 3:**
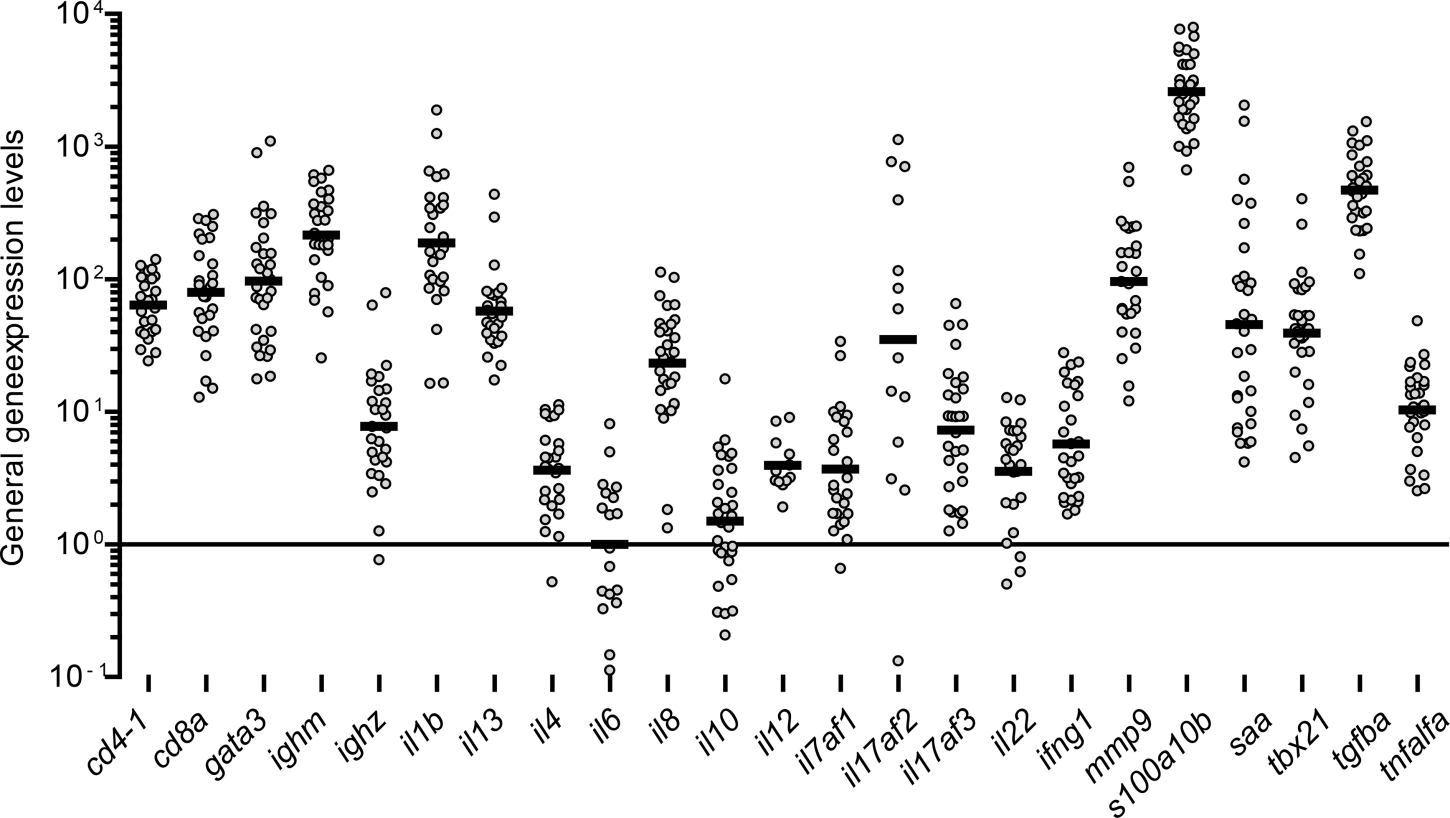
General gene expression level of the genes investigated. Samples having Cq values (all groups) are presented. The relative gene expression levels of the individual genes was calculated by 2^-ΔCq^ followed by normalization to the geometric mean of *il6*, which had the lowest expression level. The black lines indicate geometric means. The gene *s100a10b* had the highest overall expression level by having a geometric mean 1019 folds higher than *il6*.

**Fig 4:**
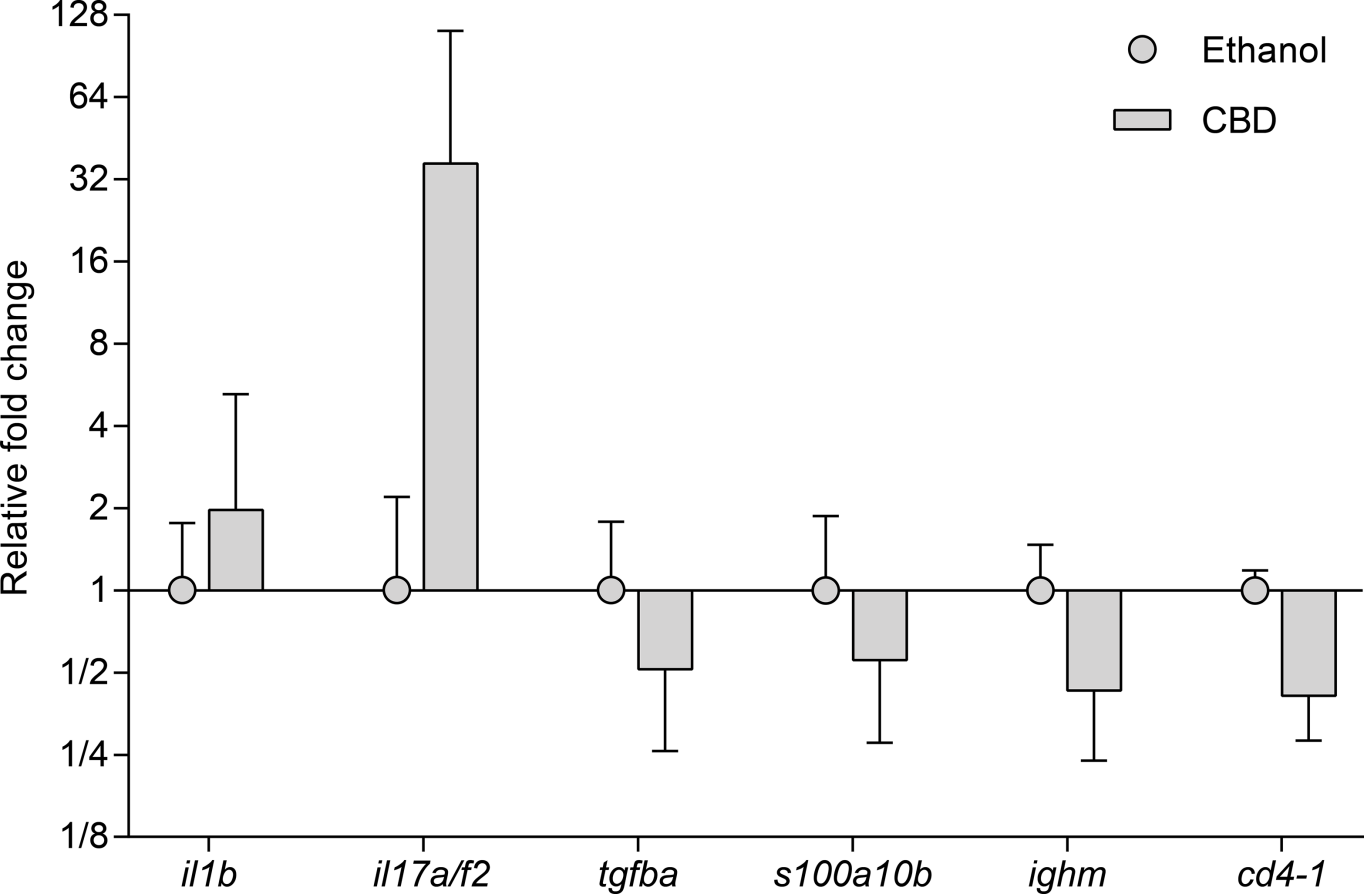
Gene expression analysis. The figure shows the geometric mean and geometric SD of the expression of 6 out of 23 immune related genes studied by quantitative real time PCR and analyzed by the 2^−ΔΔCT^ method [19]. Only statistically significant results p<0.5 (Student’s t-test) are shown. The folds (2^−ΔΔCT^) below 1 appear as fractions (e.g. 1/8 indicating 8 fold down regulation). A more comprehensive resume of all the gene expression results is available in S1 Table.

A total of 23 immune related genes were investigated. The expression level of individual genes varied in all groups and the highest expression was seen for genes encoding *s100a10b* followed by *tgfba*, *ighm* and *il1b* genes while *il22* and *il10* genes displayed the lowest level of expression (Fig 3). Significantly regulated specific genes are shown below (Fig 4) but all gene expression data are presented in the Supporting Information (S1 Table).

### Immune gene expression in zebrafish exposed to CBD compared to ethanol control fish

Two cytokine genes were significantly up-regulated following CBD exposure when compared to ethanol control fish. Thus the gene encoding *il1b* was slightly up-regulated (1.96 fold), and the gene encoding *il17a/f2* was highly up-regulated (36.52 fold). The following four genes were significantly downregulated: *tgfba* -1.93 fold, *ighm* -2.32 fold, *cd4-1*–2.44 fold and *s100a10b* -1.81 fold (see S1 Table).

### Immune gene expression in zebrafish exposed to CBD compared to water control

Three cytokine genes were significantly up-regulated compared to water control fish. Thus, the genes encoding *il1b*, *il13* and *il17a/f1* were up-regulated with 5.16 fold, 1.78 fold and 2.63 fold, respectively. In addition, the genes encoding matrix metalloproteinase *mmp9*, transcription factor *tbx21* and cellular receptor *cd8a* were up-regulated with 4.38, 2.61 and 2.15 fold, respectively.

### Immune gene expression in zebrafish exposed to ethanol compared to water control

Four cytokine genes were up-regulated in ethanol exposed fish when compared to water exposed control fish. Thus, genes encoding *il1b*, *il13*, *il17a/f1* and *tgfba* were up-regulated 2.62 fold, 1.73 fold, 2.07 fold and 1.86 fold, respectively. Two transcription factor genes were up-regulated, including *gata3* (2.15 fold) and *tbx21* (3.36 fold). Two cellular receptor genes were up-regulated comprising *cd4-1* (1.77 fold) and *cd8a* (2.33 fold). Two immunoglobulin genes encoding *ighm* and *ighz* were up-regulated 2.62 fold and 2.27 fold, respectively. Other genes that were up-regulated included *mmp9* 3.96 fold and *s100a10b* 1.62 fold.

Even though several genes were up- or down-regulated as depicted above, many did not meet our criteria of a two-fold regulation. Taking this into consideration the list of significantly regulated genes are limited to *il17a/f2*, *ighm* and *cd4-1* for CBD-exposed fish when compared to ethanol-exposed control fish. For CBD-exposed fish compared to water control fish only *il1b*, *il17a/f1*, *mmp9*, *tbx21* and *cd8a* met the criteria, and for ethanol-exposed fish compared to water control fish genes encoding *il1b*, *gata3*, *tbx21*, *cd8a*, *ighm*, *ighz* and *mmp9* were regulated.

## Discussion

The effects and impact of *Cannabis sativa* products are under discussion due to their wide usage as drugs and therapeutants [20][21]. However, many of the crude extracts and preparations of the plant contain numerous chemical constituents with different pharmacological actions (2) whereby the debate may appear un-focused. One of the major constituents (CBD) in *C*. *sativa* extracts has been studied to some extent in rodent models and cell cultures but scientific documentation of its effects is still scattered and incomplete. Epidemiological studies have not yet documented severe side effects of CBD on humans but scientific evidence from controlled experimental studies may qualify the discussion. In the present study we applied zebrafish as an experimental model to elucidate effects of CBD on behaviour and immune gene expression in a vertebrate organism. We found that adult wild-type zebrafish exposed to 40 mg/L cannabidiol for 30 min significantly altered behaviour, both when the CBD treated group was compared to fish exposed to water alone or water with solvent (ethanol). The solvent in our CBD preparation was ethanol, which is a mild irritant itself [22], but in this study we could not demonstrate any significant effect on swimming performance of fish which is consistent with previous behavioural studies on zebrafish acutely and chronically exposed to ethanol [23]. In contrast, CBD exposure clearly influenced the locomotory ability of our experimental fish. The CBD treated group exhibited a marked decrease of swimming performance (reflected as velocity and distance) when compared both to ethanol and water exposed fish.

The shorter distance moved is of course reflected in the swimming speed (velocity) of CBD treated fish (7.8 cm/sec), contrasting the water and ethanol treated fish showing velocities of 12.4 cm/sec and 11.0 cm/sec, respectively. It was not only swimming speed which was depressed by CBD. Expression of certain immune genes were down-regulated. This suggests that CBD intake, one of the main ingredients of cannabis, may affect both behaviour and immunity of higher vertebrates which should be considered when discussing CBD effects on humans. The effects may be due to a direct interaction between unaltered CBD and the host but as it is known that CBD degrades into THC in gastric fluid [24] it cannot be excluded that CBD to some extent was converted into THC in the zebrafish stomach and may confound our study on direct CBD effects on the fish. Further studies should therefore elucidate behavioural changes due to THC and CBD in a dose-response manner securing that the experimental set-up prevents degradation of CBD in the fish stomach. The present study demonstrated a distinct activation of genes encoding pro-inflammatory cytokines (il1b and il17a/f2) following CBD exposure which indicate that a certain state of inflammation was induced by CBD. *il17a/f2* expressed in zebrafish is homologous to *IL-17A* and *IL-17F* in humans [25] and acts as an inflammatory cytokine also in trout [26]. This observation is in line with upregulation of *il1b* which also is a pro-inflammatory cytokine in zebrafish [18]. In contrast other immune related genes were down regulated including *ighm* and *cd4-1*. Further, if we ignore the criteria of a two-fold regulation or higher, we see a trend for down-regulation of genes encoding immune molecules such as *tgfba* and *s100a10b* whereas *cd8a* was up-regulated. The immune regulating effect of CBD was suggested previously based on clinical trials [27] but additional detailed information about skewing of immune pathways may be obtained from further studies using the zebrafish model.

In conclusion, we saw a significant behavioural change of zebrafish following exposure to a 40 mg/L CBD solution, which significantly decreased swimming speed and swimming distance. The gene expression investigations showed an upregulation of two innate immune genes (*il1b* and *il17a/f2*) but down-regulation of several other immune related genes including *tgfba*, *s100a10b*, *ighm and cd4-1*. Our study may be partly biased due to a possible CBD degradation to THC in the experimental animal stomach. Additional behavioural and gene expression studies should therefore supplement the presented study and describe the extent of CBD degradation (if any) in the zebrafish stomach. The zebrafish model seems applicable for elucidation of cannabinoid effects on the vertebrate organism and may contribute to our understanding of *C*. *sativa* products in human patients.

## Supporting information

S1 Table Gene expression level and significance level. Green scaled numbers indicate significant gene regulation. Blue scaled Percent valid Cq indicates that Cq is below the threshold of 40%. Threshold for fold is 2. Down-regulation marked in red.
(XLSX)

S2 Table Behavioural effects of CBD on zebrafish. Raw data from the behavioural experiment and comparison of treatment groups p<0.05, Student’s t-test.
(XLSX)

S3 Table Cq values from qPCR. (XLSX)
